# Amino Acid-Induced Impairment of Insulin Signaling and Involvement of G-Protein Coupling Receptor

**DOI:** 10.3390/nu13072229

**Published:** 2021-06-29

**Authors:** Nur Fatini Zakaria, Muhajir Hamid, Mohd Ezuan Khayat

**Affiliations:** 1 Department of Biochemistry, Faculty of Biotechnology and Biomolecular Sciences, Universiti Putra Malaysia, Serdang 43400, Selangor, Malaysia; fatinizakariacontact@gmail.com; 2 Department of Microbiology, Faculty of Biotechnology and Biomolecular Sciences, Universiti Putra Malaysia, Serdang 43400, Selangor, Malaysia; muhajir@upm.edu.my

**Keywords:** insulin resistance (IR), type 2 diabetes mellitus (T2DM), branched-chain amino acid (BCAAs), metabolic dysfunction, gut microbiome, inflammation, mammalian target of rapamycin (mTOR), G-protein coupled receptor (GPCRs)

## Abstract

Amino acids are needed for general bodily function and well-being. Despite their importance, augmentation in their serum concentration is closely related to metabolic disorder, insulin resistance (IR), or worse, diabetes mellitus. Essential amino acids such as the branched-chain amino acids (BCAAs) have been heavily studied as a plausible biomarker or even a cause of IR. Although there is a long list of benefits, in subjects with abnormal amino acids profiles, some amino acids are correlated with a higher risk of IR. Metabolic dysfunction, upregulation of the mammalian target of the rapamycin (mTOR) pathway, the gut microbiome, 3-hydroxyisobutyrate, inflammation, and the collusion of G-protein coupled receptors (GPCRs) are among the indicators and causes of metabolic disorders generating from amino acids that contribute to IR and the onset of type 2 diabetes mellitus (T2DM). This review summarizes the current understanding of the true involvement of amino acids with IR. Additionally, the involvement of GPCRs in IR will be further discussed in this review.

## 1. Introduction

Ample amounts of basic macromolecules such as carbohydrate, protein, and fat are crucial for optimum bodily function. Nevertheless, augmentation in any of these particular food categories may result in various detrimental effects. Insulin resistance was proven in numerous studies to be the onset of many metabolic disorders in humans, especially diabetes mellitus [1,2]. An elevated concentration of certain plasma amino acids was intricately linked as one of many diverse factorial precursors in the development of diabetes. Branched-chain amino acids (BCAAs) have been found to be escalated in people with insulin resistance, and therefore may serve as possible biomarkers in the early detection upon onset of the disease [3,4,5].

Over the last few decades, the pervasiveness of diabetes has surged across the globe. The status of diabetes has changed from being classified as a subdued disorder of elderly people to being a major cause of morbidity and mortality affecting the youth and middle-aged people [6,7]. According to the Diabetes Atlas 9th edition 2019, published by the International Diabetes Federation, there are around 462 million people living with diabetes globally, and the number is expected to rise unless urgent preventive measures are taken. Type 2 diabetes mellitus (T2DM) accounts for 85% of total diabetes [6] and is known to appear later in life. This phenomenon yields damaging consequences on a nation’s health and economy if left untreated [7]. T2DM is characterized by the amount of blood sugar/glucose (mg) per deciliter (dL) of blood. Normal blood sugar levels measured with a fasting blood glucose test are ≤99 mg/dL, while any reading greater than 125 mg/dL is associated with a higher risk of developing insulin resistance, or worse, diabetes [8]. There are numerous etiologies associated with T2DM, but the one factor widely known to ignite this metabolic disorder is insulin resistance.

Insulin resistance indicates the inability or inefficiency of the body to respond well to hormone insulin [9]. The pancreatic islet of Langerhans consists of three types of cells, i.e., alpha cells, beta cells, and delta cells. Peptide hormone insulin is secreted by β-cells of the islet, which function as messengers that instruct designated peripheral insulin-sensitive cells in the body to take up glucose [10]. Insulin binding to its respective insulin-sensitive cell activates a series of downstream signal transduction responses in cells, which will eventually recruit GLUT4 translocation to the membrane of cells. GLUT4 enables glucose to influx into cells [11]. Any defect that interferes with this process would potentially promote malfunction of the pancreas, because constant ectopic production of insulin is needed to compensate lower blood sugar levels [10]. Persistent occurrence of this incident leads to permanent impaired glucose tolerance/prediabetic, which, if left unchecked, leads to insulin resistance. Retinopathy, neuropathy, neointimal hyperplasia, microvascular and macrovascular disease, permanent pancreas dysfunction, among others, are the complications and paradoxical effects that coincide with diabetes [6,12].

Certain amino acids were observed to be particularly elevated in T2DM. Branched-chained amino acids (BCAAs) and aromatic amino acids (AAAs) were shown to be associated with insulin resistance and may serve as possible markers prior to the onset of diabetes. Upon comparison with the metabolic marker homeostatic model assessment of insulin resistance (HOMA-IR) for diabetes, recent studies provide evidence that BCAAs and AAAs are closely correlated with the progression of T2DM [2,4,5,13,14,15]. Recent studies constructed regarding this event give rise to several probable mechanisms concomitant to this situation. BCAAs are essential amino acids that include leucine, isoleucine, and valine and account for 40% of total amino acids needed by mammals [16,17]. The isobutyl group presents branching in the side chain of leucine and isoleucine at γ- and β-carbons, respectively [12]. The tampering of BCAAs in the impairment of insulin action was broadly reviewed as a consequence from the downregulation of BCAA metabolism in muscle controlled by the mTOR pathway, which is known to be regulated by insulin and leucine. Interference with this pathway is seen to culminate in insulin resistance. There are also studies which articulate that the elevation of BCAAs over a period of time is causal and contributes to inflammation, which will eventually result in insulin resistance [2]. 

The direct and accurate elucidation correlating to the actual involvement of AAAs with the impairment of insulin action still demands more research. AAAs are strongly associated with obesity-related impaired insulin sensitivity patients [18,19]. The causality correlating to this event is from the dysregulation of amino acid metabolism. A study in 2016 examined the global transcript profile of adipose tissue and skeletal muscle; the peripheral cells and the microarray analysis results revealed the upregulation and downregulation of certain genes. Upregulated genes are involved in the adaptive immune response, which scans for the inflammation and infiltration of inflammatory cells, both of which induce insulin resistance over time. The downregulated genes are those responsible for the metabolism of AAAs, which evidently explains the etiology behind the elevated plasma concentration of AAAs in insulin resistance/T2DM patients [14]. 

Faulty insulin receptor activity often breeds many metabolic complications in consideration that insulin influences the majority of cellular metabolism processes. Moreover, metabolic disorders such as insulin resistance are eminently known to be provoked through impaired action of this receptor [9,20]. At present, the G-protein-coupled receptor (GPCR) highlights its ability to induce the insulin signaling pathway independent of insulin perception, which is mediated through cross-talk with the insulin receptor [21,22,23,24]. Nutrient-sensing receptors T1R1/T1R3, T1R2/T1R3, GPRC6A, and CaSR are class C GPCRs, possessing the capability of detecting AAs. A detailed review on the association of amino acids with the prevalence of insulin resistance is presented in this review.

## 2. Normal Insulin Action/Insulin Action in Normal Settings

Insulin is a peptide hormone known to strongly mediate its metabolic action on liver, skeletal muscle, and adipose tissue. The insulin signaling pathway is critical, and needs to be kept in homeostasis at all times to ensure general well-being. Following the ingestion and digestion of food, β-cells of the islet of Langerhans sense the elevated blood glucose level and secrete insulin. The various metabolic actions of insulin include increased glucose uptake, increased glycolysis and glycogen synthesis, increased lipogenesis, increased amino acid uptake and protein synthesis, decreased gluconeogenesis in the liver, decreased glycogenolysis, lipolysis, and protein degradation at peripheral tissues, mainly the skeletal muscle and adipose tissue [25]. The insulin signaling pathway consists of three parts: (1) binding of insulin to its cognate receptor; (2) the signal transduction pathway; and (3) response through the transport of glucose across the cellular membrane.

**Figure 1:**
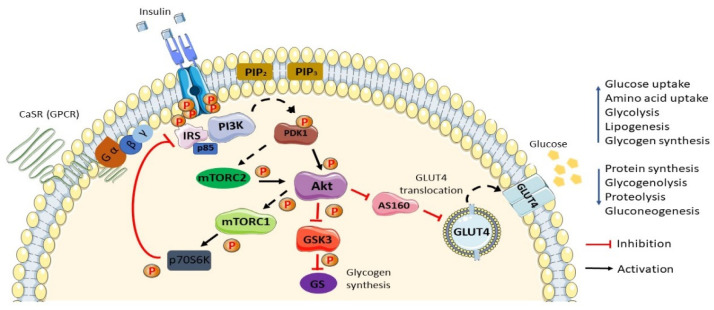
Schematic diagram of the general insulin signaling pathway in cells mediated through IR/RTK. The phosphorylation of RTK recruits various downstream regulatory proteins. The insulin signal is transduced among the target proteins/enzymes until GLUT4 vesicle fusion with the plasma membrane of the cell: CaSR, calcium-sensing receptor; IRS, insulin receptor substrate; p85, phospho-PI3 kinase *p85*; PI3K, phosphoinositide 3-kinases; PIP2, phosphatidylinositol 4,5-bisphosphate; PIP3, phosphatidylinositol 3,4,5-bisphosphate; PDK1, phosphoinositide-dependent protein kinase-1; mTORC (1/2), mammalian/mechanistic target of rapamycin complex (1/2); Akt, protein kinase B; AS160, Akt substrate of 160 kDa; GLUT4, glucose transporter 4; GSK3, glycogen synthase kinase; GS, glycogen synthase; p70S6K, ribosomal protein S6 kinase beta-1 (S6K1); P, phosphate. Phosphorylation activation (black arrow). Phosphorylation inhibition (red arrow).

The insulin signaling pathway (Figure 1) begins with the binding of the peptide hormone insulin to its corresponding receptor, the insulin receptor. The insulin receptor is a receptor tyrosine kinase (RTK) that conformationally consists of two alpha and two beta subunit tetramers [26]. The insulin receptor exhibiting kinase activity is responsible for its autophosphorylation at the tyrosine residue site upon insulin binding. Activation of the receptor results in the recruitment and cascade of phosphorylation activation of many enzyme/protein molecules liable to its signal transduction pathway [27]. The insulin receptor substrate (IRS), mainly IRS-1/2, includes adaptor protein isoforms subjected to provide a docking site for PI3K enzyme binding capable of propagating the downstream signal of insulin [28]. The phosphorylation activation of PI3K, a serine/threonine protein kinase, is essential for the conversion of phosphatidylinositol 4,5-bisphosphate (PIP2) to phosphatidylinositol 3,4,5-trisphsphate (PIP3) in the plasma membrane. PIP3 is a second messenger vital for the amplification of insulin signals, and an upsurged concentration of PIP3 recruits phosphoinositide-dependent protein kinase 1 (PDK1), which will consequently activate Akt/PKB (protein kinase B) [29]. The activation of Akt inhibits regulatory AS160, bringing about the translocation of the GLUT4 vesicle reservoir in the cytosol to the membrane of the cell to take up glucose [30]. This signaling pathway is critically important for the maintenance of normal blood glucose levels.

## 3. Insulin Resistance and Type 2 Diabetes Mellitus

**Figure 2:**
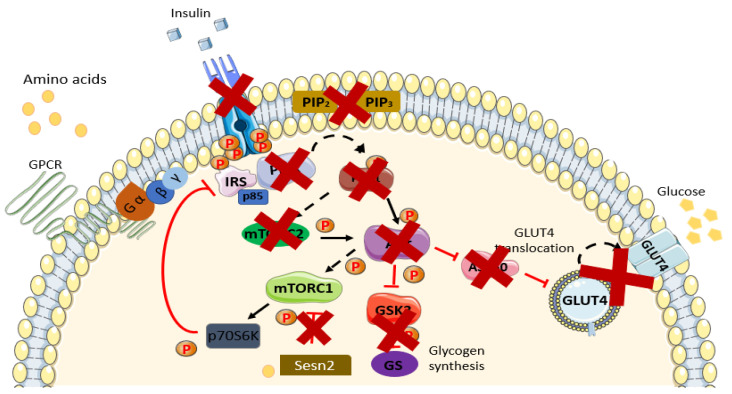
Schematic diagram of the insulin-resistant state. Available peptide hormone insulin failed to activate IR/RTK. Downstream signaling of the insulin signaling pathway is blunted in the insulin-resistant state and in the prevalence of T2DM. Augmentation in AAs in the insulin-resistant state further exacerbates the condition via activation of the mTORC1 pathway through AA (leucine) binding with Sestrin2. Activation of the mTOR pathway induces phosphorylation inhibition on serine residues of IRS1/2 and causes insulin resistance. A more thorough explanation of the involvement of AAs is found in the review. Sesn2, Sestrin2.

Insulin resistance (IR) is the leading pathophysiological factor that contributes to type 2 diabetes mellitus (T2DM) and is spreading rapidly all around the globe [31]. IR is characterized by the inefficiency and inability of insulin-sensitive peripheral tissues to respond normally to insulin action [32] (Figure 2). Consequently, this circumstance triggers compensatory action by β-cells of the pancreas in order to maintain a normal euglycemic state. Prolonging the occurrence of this event eventually leads to permanent β-cell dysfunction [9]. Notably, there are various factors recognized to trigger insulin resistance: for instance, obesity [27,33,34,35,36], critical hyperglycemia [7,37], low-grade inflammation [34,36], the overproduction of reactive oxygen species (ROS) [37,38], mitochondrial dysfunction [39,40], impaired insulin signaling pathway [41], short-chain fatty acids (SCFAs) [42], and amino acids [2,4,40,43,44]. Elevated plasma amino acid concentration has been proven in many studies to be the biomarker and causality in the development of IR. One study by Jenganathan et al. showed that amino acids are implicated with IR, although this is rather temporal and reversible. The incubation of myotubes with amino acids and insulin significantly increases the level of phosphorylation and activation of the mTOR pathway; however, this is reversible as well [45].

T2DM is a complex metabolic disorder which is well known to evolve later in life. Nevertheless, recent lifestyle advancements have altered this phenomenon; nowadays, the onset of T2DM can be observed in children and middle-aged people [7]. Early detection and preventative measures need to be addressed immediately to avert this exacerbation of T2DM, because any delayed attention could breed ugly complications. Numerous tests and analyses have been developed over the years for the detection of hyperglycemia and IR, namely, the Homeostatic Model Assessment of Insulin Resistance (HOMA-IR) [17,43,46,47,48], hemoglobin A1c (HbA1c) [48], the fasting plasma glucose (FPG) test [10,49], fasting plasma insulin (FPI) test [10], oral glucose tolerance test (OGTT) [50], intravenous glucose tolerance test (IVGTT), etc. Unchecked T2DM coincides with a myriad of complications and paradoxical effects including retinopathy, neuropathy [6,12], neointimal hyperplasia, microvascular and macrovascular disease [6,51], permanent pancreas dysfunction [10], etc.

## 4. Linkage between Amino Acids and IR/T2DM

Amino acids (AAs) are known to be particularly responsive and sensitive toward insulin action (reviewed in [12]). Elevation in the level of plasma AAs in diabetic individual is not atypical, and numerous recent studies have proven this incident [4,49,52]. Certain AAs are firmly linked to IR and T2DM, and their definite roles still need further validation and studies to determine the exact role of being an indicator or causality itself [47,53,54]. Branched-chain amino acids (BCAAs) and aromatic amino acids (AAAs) are among the AAs observed to be augmented in diabetic individuals [3,5,55]. BCAAs are the most studied and reviewed group of AAs concerning their roles in T2DM, whereas other AAs are quite limited. However, the exact mechanism causing IR and T2DM remains uncertain; several mechanisms are known to be contributing factors.

Branched-chain amino acids (BCAAs) valine, leucine, and isoleucine are essential amino acids with branching at their R-group, which need to be consumed in moderation by the human body due to its inability to synthesize them. Among other AAs, BCCAs are the most studied group of AAs recognized to be augmented in IR and T2DM [13,56]. Many studies have deduced numerous possible mechanisms regarding the events contributing to the onset of IR and eventually T2DM. Conversely, several studies have illustrated the health benefits of the dietary consumption BCAAs, including enhanced insulin sensitivity, improvement of glucose disposal, enhanced muscle development, obesity management, and an improvement in overall health when coupled with an active lifestyle [15,17,18]. This unparalleled jurisdiction should be addressed more attentively in seeking the real underlying advantages and disadvantages of dietary BCAAs to one’s health. In summary, reviewing such extensive studies upon BCAAs, several cellular mechanistic actions did come to light.

### 4.1. Metabolic Dysfunction

In some studies, results linked BCAAs and IR due to impairments in the catabolic pathway. Certain enzymes attributed to BCAAs catabolism are observed to be downregulated, thus interfering with complete BCAA metabolism. The first two catabolic steps engage the identical actions of two enzymes. Isoenzyme branched-chain amino acid aminotransferase (mitochondrial BCAT and cytosolic BCAT) catalyze the first reversible transamination/deamination process of BCAAs into their respective α-keto acids, which is coupled with the conversion of α-ketoglutarate into glutamate. Leucine, isoleucine, and valine yield α-ketoisocaproate (KIC), α-keto-β-methylglutarate (KIM), and α-ketoisovalerate (KIV), respectively. Irreversible oxidative decarboxylation, the second step of BCAA catabolism, utilizes the catalytic activity of the branched-chain keto acid dehydrogenase (BCKD) complex. Branched-chain acyl-Coenzyme A (CoA) species were produced respective to their cognate α-ketoacids. A complete metabolism of BCCA species generates anaplerotic metabolites of TCA cycle function for the subsequent fuel generation process (ATP), lipogenic, ketogenic, or glucogenic substrates [12,43,57,58,59]. Elucidation concomitant to the impairment of BCAA catabolism was demonstrated in several studies as a repercussion to the downregulation of pivotal genes necessary for BCAA catabolism [32,60]. An outstanding study by Kirsi et al. on monozygotic twins supports this statement; a significant downregulation of BCAA catabolic genes (*BCAT2*, *BCDKHB*) was observed in obese co-twins. Reduction in the catabolic metabolites of BCAA species further justifies the observed data [61,62]. A cross-sectional study in 2016 also reported on the remarkable downregulation of genes with positive correlation with IR modulating the BCAA metabolism. *BCKDHB*, *DBT*, *DLD*, *IVD*, *ACADSB*, *MCCC1/2, ECHS1*, *EHHADH*, *ACAA2 HMGCS2*, *OXCT1*, *ALDH6A1/ALDH3A2*, *PCCA/B*, and *MUT* were among the proximal, middle, and distal genes of the metabolic pathway noted to be significantly reduced. These genes were positively correlated with insulin insensitivity/IR [63]. Additionally, prominent downregulated BCAA catabolic genes in the adipose tissue of subjects with elevated BCAA serum concentrations were correlated with high HOMA-IR values (*p* < 0.05). Similarly, from the Kyoto Encyclopedia of Genes and Genomes (KEGG) enrichment analysis, there was an outstanding discovery of the most-downregulated pathways (*BCAT1*, *BCKDHB*, *MCC2*, *AUH*, *PCCA*, *PCCB*, *HIBADH*, *ALDH6A1*): the BCAA degradation pathways (*p* = 1.1 × 10^−7^) [14]. In line with others, an assessment of muscle biopsy data between normoglycemic and hyperglycemic individuals displayed distinct contrast to the upregulation of the *PMK1* (encoded BCKD phosphate subjected to BCKD activation) gene, which is a rate-limiting step of the complete metabolism of BCAAs. *PMK1* was upregulated in normoglycemic subjects and vice versa [64]. Evidence from the studies reviewed reinforces that the cellular mechanism of the impairment of BCAA catabolism is due to the downregulated genes of its metabolism, thus inducing a higher BCAA level, which is causal to the prevalence of IR/T2DM.

### 4.2. mTOR Pathway

Leucine influences impairment in the insulin signaling pathway through negative loop phosphorylation inhibition on the IRS serine residue of the mTOR pathway, and it has been heavily investigated and reviewed by many studies [12,18,40,65,66,67]. The mechanistic/mammalian target of rapamycin (mTOR) is a pathway responsible for the regulation of various vital cellular responses such as cellular metabolism, and it is closely associated with the insulin signaling pathway (reviewed in [68]). mTOR, a serine/threonine kinase, exists in two distinct complexes: complex 1 (mTORC1) and complex 2 (mTORC2); each account for a different function. mTORC1 exhibits mechanistic ability to negatively regulate the insulin signaling pathway through the phosphorylation inhibition of IRS serine residue mediated through the action of ribosomal protein S6 kinase 1 (S6K1) [13,58,68,69]. Inhibition of IRS inhibits the binding and activation of PI3K, causing impaired insulin signaling. Leucine activation of mTORC1 is mediated through direct binding to Sestrin2 [70]. As discussed earlier, it is well known that in an IR state, BCAA catabolism is impaired and downregulated. A study by Sonja et al. on fibroblasts with monogenetic defects in leucine catabolism demonstrated significant S6K1 activation in comparison to controls. Additionally, maximum S6K1 activation was seen to be consistently higher in defective strains even at defined leucine concentration [58]. HF (high fat), HF/BCAA, and SC (standard chow) diets fed to rats displayed clear increments in the phosphorylation of mTOR, S6K1, and IRS in the HF/BCAA group. The mechanistic approach in regard to mTOR mediating IR was further supported through the inhibition of mTORC1 via rapamycin, because the effect was reversed [18]. Xiao et al. analyzed the effects of the deprivation of individual AAs on the insulin sensitivity and metabolism of glucose, and they reported that hepatic insulin sensitivity was restored when subjected to leucine deprivation. Following leucine distress, insulin signaling is presumed to be reverted to normal, supporting the previously observed data [56]. A controlled concentration of AAs shows acute activation of the mTORC2 and AMPK pathway, which is sensitive to autophagy, highlighting that the mindful consumption of BCAAs promotes autophagy and the cell renewal process [69]. Western blot analysis of the liver illustrated a significant reduction in mTORC1 activity in PD (protein diluted) subjects and its attenuation during BCAA repletion [71]. In contrast to mTORC1 activation igniting IR, exercise (RE) is also known to activate mTOR but deliver rather different results [72,73]. Therefore, the discrepancy between both events still demands further validation. Bae et al. validated the effect of exercise on diabetic mice to improve insulin sensitivity and glucose uptake into cells. Nevertheless, exercise-induced mTOR activation in mice indicated a lower activation of mTORC1 and higher mTORC2 and p-Akt proteins level, suggesting the possible amendment of IR via the action of mTORC2 [74]. A study in 2018 highlighted the mitigation of IR through exercise mediated through MG53 regulation. db/db mice displayed a lower level of MG53 and improved insulin sensitivity after exercise in correspondence to controlled diabetic mice [75]. A transparent causal effect of BCAAs through the activation of mTORC1 is mechanistically conclusive to the onset of T2DM [72,73,74,76].

### 4.3. Gut Microbiome

The gut microbiota plays a major role in modulating the host’s health condition; they function as the second organ of the host. Symbiotic relationships among microorganisms residing in the host gastrointestinal (GI) tract are crucial for the maintenance of metabolic homeostasis in the body. The gut microbiota, comprising over ≈100 trillion bacteria in the human intestine, has been proven by several studies to influence obesity and insulin resistance dependent upon its composition [77,78]. Prominent phyla often found in human and rat guts are the *Bacteroidetes* and *Firmicutes*, and modification of their ratio influences the overall body metabolomic processes [1]. In healthy and lean subjects, higher numbers of *Bacteroidetes* in relation to *Firmicutes* are frequently observed [79,80]. Previous studies have suggested that the gut microbiota may influence the catabolism of protein and circulating BCAAs in the body. For instance, obese animal models displayed augmentation in intestinal *Firmicutes* and fewer *Bacteroidetes* [77,81]. Interestingly, apart from its influential traits, previous studies have demonstrated the ability of gut bacteria to synthesize AAs through de novo biosynthesis [82,83]. Notably, because epidemiological study has attributed substantial links between BCAAs and IR, a recent study in 2019 on the restriction of dietary BCAAs displayed positive results. Subjects with diabetes showed improvements in insulin sensitivity despite lower meal-induced insulin secretion, increased insulin-sensitizing hormone FGF21, a 1.7-fold increment in mitochondrial activity, lower fasting plasma BCAA levels, and the expression of mTOR [1]. Adjustment of the host dietary protein consumption portrayed rapid modification of the composition of gut microbiota. Observation of the fecal microbiomes of diabetic subjects after dietary intervention showed them to be enriched with *Bacteroidetes* [1,78]. A recent study by Yoriko et al. investigated the manipulation of gut microbiota-dependent metabolites and their precursors in order to improve the overall metabolic conditions over the course of 2 years [78]. Reduction in the precursor and gut microbiome-dependent metabolites significantly improved glucose tolerance and insulin sensitivity [84]. Alternatively, Pedersen et al. presented that the altered composition of gut microbiota impacts the complete catabolism of BCAAs and insulin resistance, thus highlighting the independent contribution to the elevation of BCAAs [85]. Lipopolysaccharide (LPS) is the outer coat of Gram-negative species of the gut microbiota, and is known to increase intestinal permeability and is suggested by some studies to trigger inflammation and further aggravate IR and T2DM [77,86]. On the contrary, lipopolysaccharide-binding protein (LBP) that binds to LPS in a BCAA-enriched mixture (BCAAem) displayed a significant reduction in LPS levels (*p* < 0.05) [87,88]. BCAA supplementation in middle-aged mice presented positive changes and altered the gut microbial composition. Dietary intervention with BCAAem displayed beneficial effects such as delayed gut microbiome shift due to aging, because the rate of adjustment in the *Bacteroidetes* to *Firmicutes* ratio was slower and increased the lifespan [88,89]. Therefore, the connection of the gut microbiome communities is deemed as both a cause and an indicator of IR in relation to AAs.

### 4.4. 3-Hydroxyisobutyrate (3HIB)

3-hydroxyisobutyrate (3-HIB), a downstream catabolic metabolite of valine, is a paracrine regulator of fatty acid species transport, and its accumulation in skeletal muscle mechanistically induces IR [54]. Although a surplus of lipid species in skeletal muscle breeds IR, its transport into muscle is still inconclusive. PGC-1α (*Ppargc1a*) in skeletal muscle manipulates various genetic process such as mitochondrial biogenesis, fatty acid metabolism, and the activation of angiogenesis; in a paracrine manner, it is also in control of lipid species transport and oxidation to mitochondria. 3-HIB is derived from 3-hydroxyisobutyryl-coenzyme A (HIBC) via the action of hydrolase enzyme (HIBCH). Impaired valine catabolism in IR induces the elevation of 3-HIB, which further worsens the situation. Skeletal muscle of mice treated with 3-HIB showed an accumulation of triacylglycerides (TAG) and diglycerides (DAG), which are famous contributors to blunted insulin action/IR through RTK inhibition via PKC-θ. In one study, an accumulation of DAG in skeletal muscle was in line with higher PKC-θ phosphorylation activation species. Blunted insulin response induced by 3-HIB and DAG was further proven with lower Akt activation. Notably, supplementary to high 3-HIB levels, muscle biopsies from diabetic mice and subjects articulated a lower expression of enzymes downstream to 3-HIB metabolism [54,90]. A significant correlation of 3-HIB with HOMA-IR (r: 0.22, *p* < 0.05) and insulin sensitivity (r: −0.26, *p* < 0.01) was observed, and lifestyle intervention incorporating hypocaloric dieting resulted in a significant reduction in 3-HIB and its effective reversal into normal settings, contradicting BCAAs [43,91]. Additionally, an accumulation of short-, intermediate-, and long-chain (C3–C16) acylcarnitine (ACs), metabolites of incomplete BCAAs, and FA oxidation, induce IR (20–30% decrease in insulin response) [63,92,93]. Perturbation in the metabolism of nutrients usually breeds other accompanying problems. The detection inability of cells sensitive to insulin generally shifts their fuel source, maintaining cellular activity through amino acids (gluconeogenesis) and fatty acids (β-oxidation) for energy production (reviewed in [66]). β-oxidation generates an abundance of acetyl-CoA, which is a precursor to the TCA cycle, and BCAA metabolism produces intermediate TCA cycle species. Impaired BCAA catabolism in IR interferes in such processes through its inability to readily provide anaplerotic species into the TCA cycle. An accumulation of AC species was supported by many studies and seen to be increased by 2–3-fold in IR skeletal muscle, suggesting defects in complete amino acid and fatty acid oxidation. Kirchberg et al. confirmed the pivotal role of BCAAs in β-oxidation in their study of infants aged 6 months experiencing higher AC values when fed HP formula milk in comparison to breast-fed infants [94]. A similar trend was seen in LGA (large for gestational age) newborns, experiencing higher AC values positively correlated with obesity and IR [92]. An accumulation of incomplete metabolized species with time is believed to cause inflammation and constantly provoke faulty processes, eventually leading to mitochondrial dysfunction [63,95]. A faulty mitochondria process and function with time would shift the IR state into T2DM [96,97].

### 4.5. Inflammation

Predictable of the interconnection of obesity and IR, evolving studies in the literature affirmed inflammation as a contributing mechanism, provoking IR [14,44,98]. A cross-sectional study on gene expression analysis in adipose tissue on high HOMA-IR (*p* < 0.05) and BCAA (*p* < 0.05) subjects disclosed the upregulation of several inflammation-related pathways. Innate and adaptive immune systems were among the upregulated inflammation-related pathways. Upregulated genes were susceptible to lysosome biogenesis and degradative enzymes, the construction of proteins required for lysosomal acidification, transport proteins, and several chemokines (IL-6). Genes encoding various immune cell species and receptors were also significantly upregulated. Meanwhile, peroxisome proliferator-activated receptor-gamma coactivator-1α (*PPARGC1A*), a crucial regulator of mitochondrial biogenesis and function, was downregulated significantly [14,39,40,99]. A study performed in 2018 reported that high BCAA concentrations induced oxidative stress and NF-κB activation, generating inflammation. Mice fed an HF diet manifested high BCAA levels, which consequently exacerbated oxidative stress and apoptosis in hepatocytes, causing permanent hepatic damage [100]. Notably, in one study, a clear influential role of BCAA was observed in mice fed with a low protein + BCAAs diet, exerting an enhanced mRNA expression of pro-inflammatory cytokines Ifn-γ, Tnf, and chemokine (all *p* < 0.05) [101]. The subsequent upregulation of pro-inflammatory genes/pathways in cells with time contributes to permanent mitochondrial and β-cell dysfunction, supporting the mechanistic action of BCAAs regulating IR. Palmitate (0.8 mM and 0.6 mM) induced insulin resistance in C2C12 myotubes, suppressed the insulin-stimulated phosphorylation of downstream target proteins, increased AA concentration (all *p* values < 0.0001), increased pro-inflammatory cytokines (TNF-α and IL6), and reduced GLUT4 mRNA expression. Inflammatory cytokines escalated the expressions of MAFbx (1.53-fold, *p* = 0.033) and MuRF1 (1.39-fold, *p* = 0.031), which are genes related to ubiquitin–proteasome pathways [14,47]. Higher proteolysis activity induced via the elevated translation of the ubiquitin–proteasomal pathway is explanatory for the higher BCAA serum concentration in IR and the T2DM state.

### 4.6. Aromatic Amino Acids (AAAs)

Aromatic amino acids (AAAs) tyrosine (Tyr), phenylalanine (Phe), and tryptophan (Trp) are known to be elevated in the blood plasma of IR and diabetic patients [3,5,17,19,47,102,103]. Tyr is the most reviewed and studied AA of the AAAs. Studies on the cellular mechanism of AAAs inducing IR are not as extensive as BCAA species. Mechanistic elaboration on the roles of AAAs and other amino acids can be considered scarce. Numerous cross-sectional and cohort studies have reported a significant positive correlation of AAAs with the incidence of T2DM. Therefore, future work on metabolic disorders should be more attentive to other AA species such as AAAs, considering their pivotal roles in metabolic disorders. 

System L-type amino acid transporters (LAT) are shared between the two species (AAAs and BCAAs) while exhibiting higher affinity toward BCCAs, and they could be one of many possible explanations of AAA serum elevation in IR incidence (reviewed in [65,67,104]). MAP4K3 is responsible for AA sensing (leucine) activating mTORC1 and is upregulated in high BCAA levels [58]. In turn, a higher influx of BCAAs would lower AAA influx into cells, which is in line with the observation of AAA species being elevated in IR incidents [105]. AAAs are precursor molecules required in the synthesis of critical neurotransmitters that control the transmission of “messages” all over the body. Catecholamines (dopamine (DA), norepinephrine, and epinephrine) are synthesized from Tyr and Phe, whereas serotonin is synthesized from Trp [106]. Major depressive disorder (MDD) is positively correlated with impaired insulin action/IR. In conditions of high BCAA and AAS levels in IR, a lower transport of AAs into cells results in lower amounts of neurotransmitter precursors and neurotransmitters. Inflammation is known to induce cellular re-programming. In line with the downturn of neurotransmitters, high C-reactive protein (CRP) and inflammatory components are observed and assumed to further aggravate the readily impaired insulin insensitivity [41,107]. A recent study in 2019 highlighted a novel postprandial circuit of tyrosine-sensitive glucose homeostasis. The study demonstrated the capability of β-cells to secrete and, in turn, bind DA in rodents. DA derived from nutritional tyrosine functions as anti-incretin and hampers hypoglycemia by inhibiting β-cells from further producing insulin/incretins. Considering its recent discovery, it is assumable that in readily impaired insulin signaling (IR), high Tyr would produce an abundant amount of DA, which, in turn, inhibits insulin production, worsening the situation [103]. Therefore, it is assumable in this situation that a high level of Tyr is dangerous to the system.

A higher level of Tyr has been implicated with higher chances of developing diabetic nephropathy (DN), which has a paradoxical effect of diabetes (OR: 0.329, 95% CI, 0.144–0.750). Chronic kidney defects are often associated with the altered synthesis, degradation, and excretion of AAAs and their metabolites. The urine of a DN subject was observed to exhibit a significantly lower Tyr level. Lower excretion of Tyr in the urine should support the reasoning behind its elevation in blood plasma [108]. Tyr can be synthesized from Phe via phenylalanine hydroxylase (PH) and its cofactor, tetrahydrobiopterin, which are both observed to be impaired in DN as the result of constant oxidative stress. Impaired PH action increases the plasma Tyr level [109,110]. Tyr can be converted into glucose in β-cells, and 3-nitrotyrosine (Tyr + free radical) has been observed to damage pancreatic islet β-cells. A faulty pancreas induced via 3-nitrotyrosine would further worsen the IR incident [111,112]. Dityrosine (Dityr), a readily oxidized tyrosine product, is implicated with mitochondrial damage in β-cells. Sprague–Dawley (SD) rats supplemented with Dityr for 24 weeks demonstrated impaired insulin sensitivity, oxidative stress of the pancreas, and inflammation. The expression of genes and transcription factors involved in the GSIS (glucose-stimulated insulin secretion) pathway (such as *Glut2*, *Gck*, *Pdx-1*, and *MafA*) was significant after the introduction of Dityr in SD rats for 24 weeks [10]. A cohort study in 2019 discussed an association of tyrosine with incidence of T2DM which was in a V-shaped manner, where any particular concentration of above µ46 mol/L was associated with increased odds of type 2 diabetes mellitus, particularly when concomitant with low high-density lipoprotein cholesterol (HDL-C) levels of 54.11 (95% CI 33.96–86.22) [113]. 

Neutrophils account for 60% of total leukocytes, which exhibit the ability to secrete AAs. Svetlana et al. (2019) discussed that in the context of their data, secreted products of neutrophils caused a subsequent shift in the plasma AA contents of individuals with metabolic disorders [114]. A 4.6-year follow-up study of 5181 Finnish men in 2019 asserted a strong correlation of AAAs with Matsuda ISI values of *p* < 5.8 × 10^−5^. Phe is subjected to decreased insulin production of the pancreas and not insulin sensitivity, whereas Tyr is significantly subjected to both [115]. Above all, Trp is observed to be associated with the impairment of insulin secretion. The kynurenine pathway predominantly (95%) metabolizes Trp-producing kynurenines, and it is acknowledged to cause excitatory neurotransmission, inflammation, and the recruitment of immune cells. Kynurenine species are confirmed to be diabetogenic to humans and inhibit insulin exocytosis by previous studies [115,116,117]. Therefore, a clear increase in Trp is explanatory for IR.

## 5. G-Protein Coupled Receptor Signaling

G-protein coupled receptors (GPCRs) are the largest plasma membrane receptor targeted by a myriad of drugs approved by the FDA [118,119]. GPCRs are characterized through their infamous seven transmembrane domain, spanning the plasma membrane with its extracellular N terminal forming complementary pockets where ligands bind, with an intracellular C terminal domain that bridges the receptor to its G-protein domains, i.e., Gα, Gβ and Gγ. GPCRs recognize a broad variety of ligands including chemicals, hormones, and proteins, portraying its fair share as an important initial regulator to virtually all physiological activities in the body. Notably, GPCRs are sub-grouped into families, i.e., (1) class A (Rhodopsin), (2) class B (Secretin and Adhesion), (3) Frizzled/Taste2, and (4) Class C (CaSR). Each group shares a similar signal activation mechanism that, upon binding of the ligand with the receptor and conformational alteration, the Gα subunit which was previously bound to GDP (inactive) now binds to GTP and dissociates itself from the complexes to start the signaling cascade. Additionally, the Gα subunits are classified into four groups with different downstream effects, i.e., (1) Gα(s), which activates the adenylate cyclase inducing the increased level of cAMP, (2) Gα(i,o), the opposite of Gα(s), (3) Gα(q/11), which activates phospholipase C (PLC), and (4) Gα(12/13), which activates Rho GTPase [23,119,120,121,122].

## 6. Involvement of G-Protein Coupled Receptor

Metabolic or glucose homeostasis are regulated by various hormones, neurotransmitters, and nutrients via GPCRs. Reassuring an unflawed cell signaling pathway is a complex process that requires the engagement of various regulatory molecules (ligands, receptors, and responses). Cross-talk between receptors has been acknowledged for decades and is required to accomplish a good signaling transduction pathway. Apart from RTK, G-protein coupled receptor (GPCR) has the ability to cross-talk with other receptors/transactivation [23,122,123]. However, few studies have been performed to prove the actual link between GPCRs, AAs, and IR. The relationship between AAs and the prevalence of T2DM are much more obvious in comparison to the direct association of GPCRs. Nevertheless, a study in 2017 suggested that GPCR angiotensin receptor type 1 (AT1R) and β-adrenergic receptors (βARs) are associated with the insulin signaling pathway through the PI3K pathway and RAS/RAF/MEK/ERK1/2 signaling cascade, respectively [124]. More studies are needed to prove the novel connection between AAs, IR, and GPCRs. GPCRs generally operate in the manner of coupling the transmembrane receptor with G-protein (Gα, Gβ, and Gγ) subunits [118]. The nutrient sensing receptor falls under class C GPCR, which is characterized by a large Venus flytrap (VFT) ligand-binding extracellular domain with obligate dimerization action (homo or heterodimer) and promiscuous ligand selection [120,125]. T1R1/T1R3, T1R2/T1R3, GPRC6A, and CaSR (calcium-sensing receptor) are class C GPCRs bearing the ability to sense AAs in the environment and execute fitting responses.

### 6.1. GPR142 and GPRC6A

The GPR142 signal through Gα(q) is a G-protein coupled receptor described by various publications to sense phenylalanine derivatives and tryptophan as its agonist and is abundantly expressed on β-cells of the pancreas. GPR142, activated by tryptophan, is a natural precursor to niacin and serotonin. The activation of this GPCR enhances glucose-stimulated insulin secretion. Recent publications have highlighted that the increased level of tryptophan in more serious IR subjects displayed positive outcomes after inactivation of the receptor knockdown in mice, and observations with its antagonist (CLP-394) have proven beneficial [126,127].

Class C GPCR, GPRC6A, responds to L-amino acids such as lysine and arginine, which bind to its large Venus flytrap N terminal extracellular receptor. GPRC6A is also seen to respond to decarboxylated osteocalcin (OCN), which improved insulin activity in wild-type mice while providing the opposite effect in *Gpcr6a*^−/−^ mice. This receptor is reproduced in numerous animal species [128].

### 6.2. Umami Taste Receptor T1R1/T1R3

T1R1/T1R3 (umami taste receptor) and T1R2/T1R3 (sweet taste receptor) have been demonstrated by many studies to activate Gαi (α-gustducin) and are broadly accessible all over the body rather than only restricted to the digestive track. After its discoveries, T1R2 and T1R3 have been intimately assigned to potentially regulate nutrient uptake and digestion. Consequent to dietary carbohydrate and protein consumption, a higher expression of T1R3, T1R2 and α-gusductin is noticed in conjunction with the available nutrients [129,130]. In pancreatic β-cells, AA binding to T1R1/T1R3 stimulates the MAPK ERK1/2 pathway, which is known to mediate insulin expression and exocytosis via the nutrient-dependent insulin gene transcription. Taste perception of T1R1/T1R3 and T1R2/T1R3 results in incretins (GLP-1 (sweet nutrients) and cholecystokinin (umami/AAs)) released into the GI track to further accelerate the metabolizing activity. Incretins function upon taste perception to better facilitate digestion through enhanced insulin secretion, the suppression of glucagon secretion, and overall improvements in satiety. The binding of AAs to T1R1/T1R3 synchronously activates the mTOR pathway, suggesting a probable regulatory involvement [131].

In an IR state coupled with caloric surplus and sedentary lifestyle, an increment in each dietary nutrient group (high glucose, BCAAs, AAAs, AAs, free fatty acids, incomplete metabolized products) is not uncommon. An ex vivo functional study of insulin receptors of rat taste cells in organoids disclosed that the genetic expression of proteins related to the taste cells was significantly reduced (gustducin, T1R3, SGLT, and GLUT) in an insulin-enriched environment. Reminiscent to the above notion, treatment with rapamycin did naturally reverse faulty gene expression, and normal amounts of taste cells were restored [132]. Constant and prolonged exposure toward any peculiar nutrient group would consistently result in receptor desensitization and a subsequent reduction in number [133]. Coherently, paramount to AA detection, cells with T1R1/T1R3 knockdown should perceive and detect their environment as being in an undernutrition state. Parallel to justifying the above hypothesis, cells with defects in their taste perception were observed to upregulate the genetic expression of mandatory proteins (transport proteins) to normalize the situation [134]. Therefore, it is concluded that the availability of taste receptors is governed by nutrient availability.

### 6.3. Calcium-Sensing Receptor (CaSR)

Calcium-sensing receptor (CaSR) is a class C GPCR that supervises parathyroid hormone (PTH) secretion via extracellular calcium concentration and calcium absorption at the kidney [135]. It is predominantly expressed in bone, kidney, parathyroid, and α- and β-cells of the pancreas. Apart from calcium, CaSR also responds to amino acids, and previous studies have shown that CaSR is involved in glucose hemostasis [136]. CaSR is influential in the modulation of insulin secretion/exocytosis from pancreatic β-cells via its high affinity in sensing any extracellular calcium shift [137]. Moreover, renal ischemia (RI), a complication of diabetes, was regarded to stem from an altered intracellular calcium level, and a recent study in 2018 articulated an obvious disparity observed on the CaSR, CaM, and p47phox profile between RI and diabetic STZ rats as an after-effect of inflammation. Inflammation induces a shifting of the calcium level, and this justification highlights the influential role of CaSR in T2DM [138]. In IR and diabetic states, a high concentration of AAs may worsen the readily faulty process just by being an agonist of the receptor. As discussed earlier in the paper, activation of the mTOR pathway negatively blunted insulin signaling, whereas taste perception continuously induced insulin secretion through the AMPK pathway; this significant convergence of the regulation of two pathways was sufficient to shift the onset of T2DM. In 2015, through analyses by Western blot and RTq-PCR, Xie et al. demonstrated a significantly linked association of CaSR and IR as the Akt pathway, which is a pathway related to CaSR signaling that was significantly lowered in IR rats [139]. Although evidence of the direct involvement of CaSR on amino acid-induced insulin resistance has not yet been reported, the plausibility is there, because the receptor is also activated by amino acid.

Glucagon-like peptide-1 (GLP-1), an incretin gut hormone produced by the L cells of the GI track, function to induce insulin secretion, suppress glucagon action, and improve satiety. The concentration of GLP-1 rapidly rises in blood plasma at the postprandial state. Nutrients, and, specifically in this review, AAs, regulate the secretion of GLP-1 and play a pivotal role in insulin action. AA drinks administered orally resulted in higher insulin secretion and action in comparison to AAs administered intravenously (IV) [140]. A whey protein diet improved satiation significantly due to its amino acid constituents, displaying improved insulin action and decreased hunger [141]. The above situation can be intimately linked to the nutrient-sensing ability of class C GPCRs, because the oral administration of BCAAs induces incretin secretion, which helps in overall satiation and increases insulin response, whereas intravenous BCAA does not [142]. Impaired GLP-1 secretion is expected in the IR state because abnormal plasma AAs circulate in the blood plasma [143]. The latest study in 2021 highlighted that CaSR inhibition negatively affects GLP-1 secretion and AAs differ in their ability to induce GLP-1 secretion through class C GPCRs [144]. In comparison to AAs and IR, the roles and involvement of GPCRs are still quite scarce in this study. More studies are needed to prove the actual involvements and links in order to fill the knowledge gaps here. Nevertheless, this is still a promising possible therapeutic approach in the future from the available literature on the connection between IR and the prevalence of T2DM with GPCRs.

## 7. Conclusions

The possibility of searching for the one thorough culprit and precursor that ignites IR is endless, because this metabolic disorder is complex. Nevertheless, metabolic advancement in the last few years has dismantled the intimate involvement of AAs being the cause and biomarker of IR. Among other AAS, BCAAs have been particularly famous and heavily reviewed by numerous studies. BCAAs account for 40% of the total AAs required by mammals, which explains its significant involvement in metabolic disorders [3]. Feedback mechanisms are the adaptive and compensatory responses of the body to maintain homeostasis at all times. Augmentation in any particular aspect is sensed by the body, and alterations reverting the system to normal are often executed. Nevertheless, this coping mechanism performed by the body must be coupled with the subject’s lifestyle intervention, because any opposition during this time would impair and damage the system permanently. A high-fat diet or chronic calorie surplus coupled with BCAAs as proven by many studies contributes to a myriad of metabolic disorders such as obesity and IR [20,66]. Mechanistic explanation on the causality of AA species breeding IR is extensive. Therefore, future studies that focus on other AA species should be more attentive, considering their significant correlation to established metabolic markers. As deliberately discussed in the paper, it is now unambiguous that AAs are both markers and causes of IR.
